# A challenging case of COVID-19: a COVID-19 positive adolescent presented with severe diabetic ketoacidosis, resistant hypertension

**DOI:** 10.1186/s12902-022-00979-8

**Published:** 2022-04-05

**Authors:** Reza Najafi, Nahid Mamizadeh, Seyed Hossein Hosseini, Sima Roushenas, Leila Bazhdan

**Affiliations:** 1 Department of Pediatric Endocrinology and Metabolism, School of Medicine, Ilam University of Medical Sciences, Ilam, Iran; 2 Department of Pediatric Nephrology, School of Medicine, Ilam University of Medical Sciences, Ilam, Iran; 3 School of Medicine, Ilam University of Medical Sciences, Ilam, Iran; 4 Student Research Committee, Shiraz University of Medical Sciences, Shiraz, Iran; 5 Department of Pediatrics, School of Medicine, Emam Khomeini Hospital, Ilam University of Medical Sciences, Ilam, Iran

**Keywords:** Case report, COVID-19, Diabetes mellitus, Diabetic ketoacidosis, SARS-COV-2

## Abstract

**Background:**

Severe acute respiratory syndrome coronavirus 2 (SARS-CoV-2) is a virus which causes COVID-19. It binds to the angiotensin-converting enzyme 2 (ACE2) receptors, expressed in key metabolic organs and tissues, including pancreatic beta cells, adipose tissue, the small intestine, and kidneys. This condition has been linked to a variety of additional symptoms, including acute encephalopathy, changes in consciousness, and even gastrointestinal bleeding.

**Case presentation:**

In this study, we have reported a 13-year-old boy, 69 kg, with SARS-COV-2 infection. In this case, multiple systems, including the endocrine, renal, pulmonary, gastrointestinal, and nervous systems, were affected.

**Conclusions:**

It is speculated that different manifestations of COVID-19 can be seen in clinical settings, and practitioners should be more cautious not to miss the chimeric characteristics of COVID-19 infection.

## Background

Novel coronavirus pneumonia was first described as the pneumonia of unknown cause in Wuhan at the end of 2019 and then became pandemic. Over time, some new extrapulmonary manifestations of this viral pneumonia were described [1–8]. While most mild instances had ordinary cold symptoms, COVID-19 is associated with multiorgan failure in severe individuals [9]. It may occur together with diabetic ketoacidosis (DKA) as the earliest manifestation of DM. Kidney damage is common in COVID-19, including proteinuria and hematuria to acute kidney injury (AKI). COVID-19-associated AKI is a risk factor for all-cause in-hospital death in patients with COVID-19 [6]. Regarding the increase in the number of patients and the lack of dialysis units in hospitals, the management of these cases seems necessary [10].

GI manifestations include nausea, vomiting, diarrhea, abdominal pain, and GI hemorrhage [5, 11]. Gastrointestinal bleeding (GIB) was reported in 2–13% of patients hospitalized with novel coronavirus disease 2019 (COVID-19).

Neurologic complications are prevalent in COVID-19 patients who are hospitalized. Rates vary depending on research methodology and patient factors, although myalgia, headache, loss of consciousness, and encephalopathy may be prevalent, affecting around one-third of patients [2, 3]. In this case report, evaluating a patient who has not previously had endocrine, neurological, and renal problems which has developed them in terms of Covid 19 was considered.

## Case presentation

A 13-year old boy, 69 kg in weight, was brought to the emergency department in Imam Khomeini Hospital, Ilam, Iran, on November 12th, 2020, with a decrease in consciousness level and respiratory distress. He was admitted with the impression of encephalopathy and COVID-19. A 10-day history of nausea and non-bloody vomiting, anorexia, polyuria, and polydipsia was provided. During this time, the patient’s diet was limited to juice and water. Furthermore, a weight decrease of 10 kg was seen throughout this time frame. The dyspnea and hoarseness had started 4 days before admission, and he had Kussmaul respiration 1 day before admission. The patient had become irritable and had malaise, headache, and disorientation to time, place, and person.

All individuals in his family were COVID-19 positive, and he had close contact with them at home. One week before his admittance, his family began self-treatment with azithromycin and co-amoxiclav. On admission, the vital signs are as follows: blood pressure (150/90 mmHg), pulse rate (128 bpm), respiratory rate (30 bpm), temperature (37 °C), oxygen saturation (95%). He had a body mass index (BMI) of 30 kg/m^2^. An electrocardiogram (ECG) showed sinus tachycardia, and other findings were normal. The blood sugar was 481 mg/dL during arrival, and venous blood gas analysis was as follows: pH: 6.92, PCO2: 24.7 kPa, PO2: 40 kPa, base excess (BE): − 27.7, bicarbonate level: 5.1 mEq/L. Initial urinalysis on the day of admission is mentioned in Table 1. He had a high anion gap metabolic acidosis on the first day (AG: 36.9, Cl: 95, Na: 137, k: 4.8 mmol/L). On the 1st day of admission, Kidney function tests were Cr (1.5 mg/dL) and urea (45. 4 mg/dl). Ten days later, Cr (4.1 mg/dL) and urea (141 mg/dL) were reported, as noted in Table 2.

**Table 1:** Results of complete blood cells (CBC), urine analysis (U/A) and erythrocyte sedimentation rate (ESR), and C-reactive protein (CRP)

	12 Nov.	14 Nov.	16 Nov.	19 NOV.	21 NOV.	23 NOV.	Ref.
CBC							
WBC10^9^/l	15.7	7.7	5.1	12	13	12.	4–9.8
HB g/dl	15.5	12.3	11.4	9.3	9.7	8.6	12–15
PLT 10^3^/μl	238	178	178	278	257	269	140–440
Lymph%	12%	7%	10%	18%	24%	15%	20–50%
Neut.%	88%	90%	87%	80%	76%	82%	40–70%
U/A							
WBC	50\	30	35	8	3	N/A	NEG.
RBC	12	Many	Many	25	3	N/A	NEG.
BLOOD	+ 1	+ 3	+ 3	+ 1	+ 1	N/A	NEG.
KETON	+ 1	+ 1	Neg.	Neg.	NEG.	N/A	NEG.
PRO.	+ 2	+ 2	+ 3	+ 1	NEG.	N/A	NEG.
ESR mm/hr	12	21	N/A	10	N/A	N/A	0–15
CRP	+ 1	Neg.	N/A	NEG.	N/A	N/A	NEG.
Mg mg/dl	2.2	2.4	1.9	1.9	N/A	1.8	1.7–2.2
Phos mg/dl	3.8	3.5	2.3	3.6	N/A	N/A	3.0–4.5
Ca mg/dl	9.6	10.6	10.5	8.2	8.9	10	8.7–10.2

**Table 2:** Report of Arterial blood gas (ABG), renal function test, and electrolytes

	12 Nov.	13 Nov.	14 Nov.	15 Nov.	16 Nov.	17 Nov.	18 Nov.	19 Nov.	20 Nov.	21 Nov.	23 Nov.	Ref.
PH	6.92	7.11	7.19	7.23	7.23	7.42	7.46	7.4	7.41	7.39	7.47	7.25–7.35
PCO2 mmHg	24	15	12	17	19	19	23	18	24	20	37	35–45
HCO3	5.1	5	4.5	7.1	10.3	12.9	16.2	11.5	12	12	26.9	22–28
Na mmol/L	137	146	144	141	144	144	143	141	142	139	140	135–145
K mmol/L	4.8	4.2	3.6	3.1	2.7	3.5	3.3	4	3.6	4.6	4.2	3.5–5.5
Urea mg/dl	43	99	145	148	156	122	119	160	144	98	101	10–50
Cr mg/dl	1.5	2.1	3.5	4.5	3.5	7.6	4.5	2.7	2	1.7	1.1	0.4–1.4

The patient was tested using the polymerase chain reaction technique (PCR). SARS-COV-2 PCR test was positive. It seems that COVID-19 has caused acute kidney damage (acidosis, increased urea and creatinine, hematuria, and hypertension); hence, the patient has followed serial urea Cr and urine analysis such as noted in Tables 1 and 2. He was diagnosed with an episode of DKA in newly diagnosed DM and symptomatic COVID-19, with extra-pulmonary involvements such as an acute kidney injury.

### Severe DKA

He was admitted to PICU (Pediatrics Intensive Care Unit). Initial treatment immediately started with intravenous hydration and oxygen therapy with mask, insulin drip, and electrolytes replacement. We planned to check blood glucose (Q1h*)* and blood gas, K, and Na (Q4h). Brain edema signs and symptoms were examined every 1-h according on ISPAD clinical practice consensus, despite a reduced degree of awareness [12]. Considering the lack of a proven diagnosis of cerebral edema, no treatment was performed for it.

Based on protocol-based DKA management, the starting dose of insulin drip in DKA patients is 0.05–0.1u/kg/hr.; we started with a 0.07 insulin drip and then raised it to 0.09 u/kg/hr. 13Nov. After retrieving from severe DKA, we started insulin 1 unit/kg/day subcutaneously, but insulin had gradually increased up to 1.9 unit/kg/day due to increasing insulin dependency. Multiple factors were convincing in this case: the infection with COVID-19, acute kidney injury, corticosteroid prescription, and obesity.

### Acid-base disorder

Despite pH correction throughout DKA care, the unacceptably low level of bicarbonate [5–7] required us to administer bicarbonate after DKA leave. This latter showed that acute renal failure may be the source of this resistant acidosis. Regarding the rapid rise in Cr and urea, we had to start treatments with the impression of acute kidney injury by consecutive infusion of methylprednisolone for 6 days (1 mg per kg per 24-h), n-acetyl cysteine (NAC) 600 mg daily, and bicarbonate 20 ml (Q8h).

The case was in a critical situation, so after exiting severe DKA on 15 November, we calculated IV hydration every 6 h based on the urine output formulation [13] that brings as follows:

$$\mathrm{Insensible}\ \mathrm{water}\ \mathrm{loss}-\left(\mathrm{drugs}\ \mathrm{infusion}\ \mathrm{vol}.+\mathrm{U}/\mathrm{O}\ \mathrm{vol}.\mathrm{in}\ 6\ \mathrm{hours}.\right)$$

$$\mathrm{Insensible}\ \mathrm{water}\ \mathrm{loss}:400\ \mathrm{CC}/\mathrm{M}2/24\ \mathrm{hours}$$

$${\mathrm{m}}^2=\sqrt{\frac{hight\times wt.\kern0.5em }{3600}}$$

Patient rehydration was set up as follows: the maximum amount of fluid potentially the patient could receive was by the 9 lit/m^2^. The patient’s body surface area was 1.6m^2^; so, the maximum fluid intake per 24 h was calculated to be 6400 cc. From 12Nov to 14Nov, the fluid volume received by the patient was 6400 cc (6100 cc fluid and 300 cc for administrating injectable drugs). On 15Nov and 16Nov, the amount of fluid intake based on urine output formulation was 2100 cc and 2200 cc per day.

After starting the oral (PO) diet on 17 November, two-thirds of the liquids he drank in the last 6 h were reduced from the formulation above. The patient was not oliguric during the hospital stay.

On the first day of admission, 20 meq/lit IV KCl was prescribed, and we changed it to 30 meq/lit on the second day. Since 15Nov 5 cc KCl, 15% was prescribed every 6 h by nasogastric (NG) tube gavage as the patient was on NPO (null per os) diet. From 17Nov, the KCl order was changed to 10 meq/lit that administrated with 1/3 2/3 fluid intake (as calculated through the urine output replacement fluid). From 19Nov, his order was changed to 20 meq/lit IV KCl with 5 cc oral KCl every 12 h; that was discontinued on 21Nov. The patient went under hemodialysis on days 16Nov, 17Nov, and 19Nov.

### Resistant hypertension

Blood pressure at arrival was 150/90 mmHg. It raised to 200/110 mmHg within the in-hospital course. Then, amlodipine, Furosemide infusion, and hydralazine infusion started to control hypertension.

The patient’s mean blood pressure during the hospitalization was 128/76 mmHg, and the minimum and maximum blood pressure were 100/60 mmHg and 200/110 mmHg, respectively. The mean blood pressure of every hospitalization day is reported in Table 3.

**Table 3:** The mean blood pressure of the case in multiple days

DATE	mean BP(mmHg)
12 Nov.	130/80
13 Nov.	154/94
14 Nov.	141/88
15 Nov.	141/78
16 Nov.	138/84
17 Nov.	135/75
18 Nov.	128/73
19 Nov.	118/66
20 Nov.	120/70
21 Nov.	115/75
22 Nov.	117/72
23 Nov.	106/65

Following the blood pressure, a pediatric cardiologist was consulted, and Electrocardiogram (ECG) was requested, and it was normal.

The blood pressure returned to normal on the eighth day of hospitalization, after three hemodialysis treatments and three prescription anti-hypertension sessions. The team then opted to taper the medicine before discontinuing it. Persistent decreased level of consciousness.

The patient’s consciousness level by Glasgow Coma Scale (GCS) [14] was nine at arrival. After hemodialysis’s second session, GCS became 12; irritability and disorientation to time, place, and the person stopped. After the third hemodialysis, acidosis and uremia improved, and GCS became 15.

### Lung involvement

**Fig. 1:**
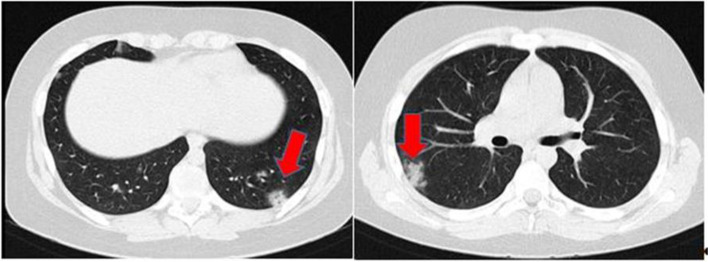
Chest CT showed bilateral peripheral and peribronchovascular patchy ground-glass opacities (arrow) in both lungs with a tree-in-bud appearance

The case had respiratory distress and tachypnea on the first day of admission. Chest CT showed bilateral peripheral ground-glass opacities in both lung fields which involved both lungs (Fig. 1). Hence, based on our national guideline of COVID-19, remdesivir was prescribed for 9 Days. He was under treatment with ceftriaxone (for possible bacterial superimposition), oxygen therapy with the mask, and chest physiotherapy. In the beginning, the oxygen saturation was 95%, and he had no decrease during the admission. He had fever episodes from 38 to 39.2 °C controlled by intravenous acetaminophen in the hospital course.

### Upper GI bleeding

On 15th November and the fourth day of admission, the patient had two upper GI bleeding episodes presented by coffee ground discharge from an NG tube. We confirmed upper gastrointestinal bleeding by submitting a sample of coffee grounds secretions to the lab, which yielded positive gastric occult blood tests. Therefore, gastric washing was done, and treatment was followed by prescription intravenous vitamin k and intravenous pantoprazole. Several minutes later, the gastric washing became clear. We reserved pack cell and FFP, sent serial CBC, and checked PT, PTT, and INR. Fortunately, lab data was normal, and there was no need to transfuse FFP or pack cells as noted results in Table 4. Fortunately, at the patient’s recent visit, the tests are normalized as shown in Table 5, and the patient’s blood glucose with insulin 1 IU / kg/day is ideally controlled for the patient’s age.

**Table 4:** Liver function test, coagulation tests, and additional renal tests

	During admission	Ref
AST U/L	18	7–55
ALT U/L	13	8–48
ALK-Ph U/L	636	40–129
Bili T. mg/dL	1.1	0.1–1.2
Bili D. mg/dl	0.5	<0.3
CPK U/L	81	*39–308*
LDH U/L	711	140–280
Alb g/dL	3.15	3.4–5.4
Amylase U/L	34	23–85
Lipase U/L	36	0–160
PT sec	13	10–13
PTT sec	27	25–36
INR	1	0.9–1.15
CL mmol/L	97.9	95–110
Anti-DNA IU/mL	1.2	< 30.0
C3 g/L	152	0.9–1.8
C4 g/L	57	
CH50 mg/dl	89.4	low:0–100 nl:101–300 hi > 301
ANA u/ml	0.7	neg. < 0.8 equ:0.8–1.2 pos. < 1.2
ANCA	0.6	neg .< 12 equ:12–18 pos. < 18
P ANCA	0.1	
Urine24h,cr mg/24 h	575	800–2000
Urine24h,pro.mg/24 h	497	
Urine24h,vol. ml	1150	600–2500
Urine24h,k meq/24 h	49.5	30–120

**Table 5:** Report of Arterial blood gas (ABG), renal function test, and electrolytes at the admission

ITEM	Result	Ref.
BUN (mg/dl)	41	15–36
Cr (mg/dl)	0.73	0.7–1.4
Na (meq/L)	135.8	135–145
K (meq/L)	4.58	3.5–5.5
FBS (mg/dl)	112	<100
HbA1c %	9.4	<5.7

### Mouth candidiasis

On the third day of admission, multiple candida lesions were inspected on the patient’s gum in physical examination, so treatment with nystatin drop started.

### C-peptide level

On 8Dec,2020, on one follow-up, fasting C-peptide was checked for the patient with RIAKEY c-peptide tube and was measured with Electrochemiluminescence immunoassay (ECLIA) method; the result was reported to be 0.2 ng/ml (normal range: 0.51–2.7 ng/ml). However, unfortunately, regarding the scarcity of related kits and severe sanctions of our country [15], autoantibodies tests were possible only in the capital (Tehran). The patient and the parents could not afford it and refused to do these tests.

### Discharge

At the discharge time, the patient was in good condition. We ordered insulin 1.2u/kg/day with a multi-daily injection method (Lantus: 27 units in the morning and 27 units night and Novorapid: 15 units before every meal of breakfast, lunch, and dinner).

### Follow-up

The patient is currently under close follow-up with the Pediatric Endocrinology and Nephrology Services. On the last visit in early July, the patient’s weight was 60 kg. The HbA1c was lower as 6.8%, and the insulin intake was reduced to 0.8u/kg/day. The renal function lab tests such as BUN and Cr were reported to be 0.9 and 18 mg/dl (both in the normal range) as noted in Table 5.

## Discussions and conclusions

We described a 13-year-old boy in weight 69 kg who experienced involvement of multiple systems due to COVID-19. These complications existed in: the endocrine system (new case of diabetes with the manifestation of diabetic ketoacidosis), renal system involvement (acute renal failure), lung involvement (seen in lung CT scan), gastrointestinal involvement (nausea and vomiting and upper gastrointestinal bleeding) and central nervous system (reduced level of consciousness).

Severe acute respiratory syndrome coronavirus 2 (SARS-CoV-2), the virus which causes COVID-19, binds to angiotensin-converting enzyme 2 (ACE2) receptors, which are expressed in key metabolic organs and tissues, including pancreatic beta cells, adipose tissue, the small intestine, and the kidneys [16].

There are two implications of these interactions. Firstly, entry of SARS-CoV-2 into pancreatic islet cells may directly induce beta-cell injury [17]. Secondly, the downregulation of ACE2 after viral entry can lead to unopposed angiotensin II, impeding insulin secretion [18]. These two factors may have led to the patient’s sudden deterioration of pancreatic beta-cell function and triggered DKA. The low level of c-peptide in the fasting phase can also be interpreted as confirmation of this hypothesis; however, we emphasize that autoantibody testing (such as islet cell antibodies (ICA) [19], antibodies to glutamic acid decarboxylase (GAD-65) [20], insulin autoantibodies (IAA) [21]) and stimulated c-peptide test) are useful in this regard, but are not available in our setting. Since the beginning of the SARS-COV-2 outbreak, multiple cases were admitted with the impression of DKA and COVID-19 [10, 17, 21, 22]. Suwanwongse et al. described three cases that developed DKA and COVID-19, while one was asymptomatic [21]. Chee et al. described a 37-years old- previously healthy case presented with DKA, provoked by COVID-19. Heaney et al. [22] reported a 54 years old male with COVID-19, DKA, and newly diagnosed diabetes. The mechanism still needs further investigation; meanwhile, there are few hypotheses in this regard. For instance, Rubino et al. [23] proposed that adherence of SARS-COV-2 to the ACE receptors of pancreatic beta cells could destroy them and induce glucose metabolism impairment. They also indicated that the abnormal immune response elicited by SARS-COV-2 might trigger an autoimmune reaction, damaging beta cells, as is expected in SARS infection (severe acute respiratory syndrome) [24]. We encountered two main problems to treat the diabetic ketoacidosis. First, recovery from the severe phase of diabetic ketoacidosis took 3days. Second, we started subcutaneously with 1 unit/kg of insulin per day. However, due to the increased need for insulin, we gradually increased the insulin to 1.9 units per kilogram per day.

As mentioned before, it was believed that this was due to the presence of active and wild infection (COVID-19), acute kidney injury, corticosteroid prescription, and obesity (BMI = 30 kg/m^2^).

Because of the reasons of acidosis, elevated urea, creatinine, persistent hematuria, and persistent hypertension from the day of admission, it is conceivable that COVID-19 had a role in the course of acute renal damage in this instance.. The kidney is not typically the main target of severe acute respiratory syndrome coronavirus 2. However, surprisingly, acute kidney injury (AKI) may occur in 4–23% of cases, whereas the dialysis management of AKI from coronavirus 2019 has not gained much attention [24]. OF course, we highlight that this is an assumption as DKA and severe dehydration can cause renal failure and not necessarily COVID-19. The prolonged lowered state of awareness following treatment of severe DKA, the incremental trend of urea and Cr, persistent metabolic acidosis, and resistant hypertension were all indicators of regular hemodialysis in this instance. Neurological complications in COVID-19 infected patients are less discussed in the literature. The patients with COVID-19 infection can present with acute encephalopathy and changes in their level of consciousness [25–29]. Neurologic complications in the patients with COVID-19 are common in the hospitalized patients. Rates vary by study methodology and patient characteristics, but myalgia, headache, and encephalopathy may be most common, occurring in roughly one-third of patients [25, 27]. To justify the patient’s decrease in consciousness level on arrival, various causes can be noticed: COVID-19 encephalopathy, cerebral edema due to DKA, uremic encephalopathy, hypertension encephalopathy, and encephalopathy due to acidosis. It is really hard to distinguish the causality here; we believe that possibly both DKA and COVID-19 [26, 28, 30] have a role in the decreased level of consciousness.

In this challenging case, we described a teenager with COVID-19. COVID-19 was associated with new-onset diabetes mellitus, severe diabetic ketoacidosis, acute renal failure, and resistant hypertension. To handle this situation, a multidisciplinary team of diverse subspecialties collaborated. We suggest that practitioners should be more careful in order to avoid missing the chimeric features of COVID-19 infection.

## Data Availability

All provided as a supplementary file.

## Abbreviations

ACE2: Angiotensin-converting enzyme 2
AKI: Acute kidney injury
DKA: Diabetes ketoacidosis
DM: Diabetes Mellitus
ECG: Electrocardiogram
ECLIA: Electrochemiluminescence immunoassay
ICA: Islet cell antibodies
IAA: Insulin autoantibodies
GAD: Glutamic acid decarboxylase
GIB: Gastrointestinal bleeding
GCS: Glasgow Coma Scale
PCR: Polymerase chain reaction
PICU: Pediatric Intensive Care Unit
SARS-CoV-2: Severe acute respiratory syndrome coronavirus 2
